# Exploring the Use of Helminthophagous Fungi in the Control of Helminthoses in Horses: A Review

**DOI:** 10.3390/ani15060864

**Published:** 2025-03-18

**Authors:** Tábata Alves do Carmo, Júlia dos Santos Fonseca, Fabio Ribeiro Braga, Adolfo Paz-Silva, Ricardo Velludo Gomes de Soutello, Jackson Victor de Araújo

**Affiliations:** 1 Department of Veterinary Medicine, Federal University of Viçosa—UFV, Viçosa 36570-900, MG, Brazil; jvictor@ufv.br; 2 Department of Epidemiology and Public Health, Federal Rural University of Rio de Janeiro—UFRRJ, Seropédica 23890-000, RJ, Brazil; juliafonseca@ufrrj.br; 3 Laboratory of Experimental Parasitology and Biological Control, Vila Velha University—UVV, Vila Velha 29102-920, ES, Brazil; fabio.braga@uvv.br; 4 Department of Animal Pathology, University of Santiago de Compostela—USC, 27002 Lugo, Galícia, Spain; adolfo.paz@usc.es; 5 School of Agrarian and Technological Sciences, Universidade Estadual Paulista—UNESP, Dracena 17900-000, SP, Brazil; ricardo.vg.soutello@unesp.br

**Keywords:** biological control, chlamydospores, nematodes, equine, nematophagous fungi

## Abstract

Equine farming faces growing challenges with helminthoses, aggravated by the indiscriminate use of anthelmintics without technical criteria. This practice favors resistance to these drugs, generates residues in animal products, compromises food safety and human health, and, when excreted in large quantities, negatively impacts environmental health by affecting invertebrates and fecal microorganisms. This highlights the importance of the One Health approach. A promising alternative is biological control with nematophagous or helminthophagous fungi such as *Duddingtonia flagrans*, *Pochonia chlamydosporia*, *Arthrobotrys oligospora*, *Monacrosporium thaumasium*, *Mucor circinelloides* and *Purpureocillium lilacinum*. Due to their different mechanisms of action, ovicidal and predatory fungi, when used together, can act in a complementary and synergistic way in the biological control of helminths, increasing their effectiveness in reducing parasitic infections. The use of these fungi through biosynthesized nanoparticles from fungal filtrates is also emerging as a new approach to nematode control. It can be administered through feed supplementation in commercial formulations. The aim of this review is to explore the use of helminthophagous fungi in the control of helminthiases in horses, highlighting their potential as a biological alternative. It also aims to understand how these fungi can contribute effectively and sustainably to parasite management in horses.

## Simple Summary

With the increase in parasite resistance to anthelmintics in horses, it is becoming essential to look for new approaches to helminth control. Helminths not only cause health problems in horses but also generate economic losses. One promising solution is the use of helminthophagous fungi, which act on the larvae and eggs of parasites. These fungi can be incorporated into horse feed, helping to reduce infestations on pasture. This review explores the potential of these fungi as an effective and sustainable tool for controlling worms in horses, promoting animal health and environmental protection.

## 1. Introduction

Businesses involving the breeding and utilization of horses play a crucial role in both developed and developing countries. The relevance of horses extends across several areas, being important for the economy, culture and science, in different social and productive contexts [1].

Horses are generally reared in extensive systems, where they are kept on pasture, almost all of which is contaminated with infective larvae and helminth eggs, which constantly contribute to helminthoses [2]. Parasitic diseases, especially those caused by endoparasites, which have a high prevalence and wide geographical distribution, are highly relevant due to their impact on animal production development [3].

Helminthiases cause direct economic losses, both in terms of treatment cost for clinical manifestations in animals and reduced production. However, due to the wide distribution of these parasites, some animals do not show any signs, and the impact of subclinical infections is more common [4]. Depending on the degree of parasite burden, helminths can cause anything from minor abdominal discomfort, weakness, and a rough coat to growth retardation, anemia and severe colic episodes, which can lead to the animal’s death [5].

Parasite control in horses on most farms is carried out exclusively using commercial antiparasitic compounds, due to their practicality, high efficiency, excellent cost–benefit ratio and ease of acquisition. However, these products are used in a suppressive and indiscriminate manner and without adequate control strategies, leading to the selection of increasingly resistant parasite populations [6].

Nematophagous or helminthophagous fungi, widely studied as natural predators of helminths, are globally distributed and naturally present in the soil, where they act as saprophytic or parasitic agents [7]. Due to their mechanism of action, fungi are classified as endoparasites, opportunists, toxin producers and predators [8,9]. The use of these fungi as biological parasite control agents is widely documented in the literature, with evidence reinforcing their potential as a crucial tool in the fight against helminthiases, helping to reduce dependence on conventional chemical control [10,11].

This review aims to explore the use of helminthophagous fungi in the control of helminthoses in horses, highlighting their potential as a biological alternative. Thus, understanding how these fungi can contribute effectively and sustainably to equine parasite management.

## 2. Methodology

This review was carried out with the aim of gathering and critically analyzing the available scientific evidence on the use of helminthophagous fungi in the control of helminthiases in horses. Given the growing concern about anthelmintic resistance and the need for sustainable alternatives in parasite management, the search for strategies based on biological control has intensified. Therefore, this study summarizes the advances in research, highlighting the mechanisms of action, efficacy and the advantages of applying these fungi in the environment and for horses, contributing to a better understanding of their potential in animal health.

A comprehensive literature review was conducted in the main databases, such as Web of Science, Google Scholar, ScienceDirect and PubMed. The search was carried out using combinations of pre-established keywords, with an emphasis on terms related to the use of helminthophagous fungi in the management of helminthiases in horses, such as ‘equine gastrointestinal nematodes’, parasite resistance to anthelmintics’ and ‘nematophagous fungi’. In addition, the keywords were adapted to align with the specific requirements of each section of the review. For the section on equine gastrointestinal nematodes, related terms were selected: ‘equine nematodes’, ‘gastrointestinal nematode control’, ‘cyathostomines’. In the ‘parasite resistance’ section, ‘macrocyclic lactones’ and ‘anthelmintic resistance’ were used. For the fungi section, keywords such as ‘biolophilic control’, ‘nematophagous fungi’, ‘duddingtonia flagrans’ and so on were used, sometimes in combination with the word ‘equine parasitosis’. In the initial stages of the writing process, 250 scientific papers were selected from the literature, covering a diverse range of formats, including literature reviews, books, research papers and articles aimed at a scientific audience. Subsequently, a bibliographic screening was carried out to eliminate articles with low relevance or published too early in the field. Research articles, reviews and clinical reports published after 2300 were selected for analysis, except for some basic theoretical studies. In addition, some articles using animals from other species were also retained for comparative reference. A total of 100 references were finally selected for this article.

## 3. Literature Review

### 3.1. Gastrointestinal Helminths in Horses

The parasite burden in horses comprises several families and genera, among which the strongylid nematodes stand out as the most prevalent. Of the 83 species described, the majority belong to the Strongylidae family and include the most common nematodes of economic importance to horses [12]. Gastrointestinal helminths in equidae have a cosmopolitan distribution across different geographic and climatic conditions [13].

Horses are among the most susceptible animal species to a wide variety of gastrointestinal parasites and can harbor several species simultaneously [14].

Strongylids are usually identified as endoparasites of the large intestine of horses, where they reach adulthood and sexual maturity. The free-living larvae develop in the pasture and the animals become infected during grazing, but they can also become infected in the stable by ingesting contaminated bedding and hay [15]. Small strongyles, also known as cyathostomes, are considered the most relevant helminths due to their prevalence, pathogenic potential and ability to develop resistance to anthelmintics [16]. These can parasitize horses of all ages, but are more pathogenic in young animals [17,18]. The most common genera of small strongyles are *Cyathostomum* spp. and *Cylicostephanus* spp. Other nematodes that affect horses are the large strongyles: *Strongylus vulgaris*, *Strongylus equinus*, *Strongylus edentatus* and *Triodontophorus* spp. In addition, there are nematodes such as *Parascaris equorum*, *Oxyuris equi*, *Strongyloides westeri*, *Trichostrongylus axei*, *Dictyocaulus*, all of which belong to the nematode phylum [19,20]. *Fasciola hepatica* is a parasite that belongs to the class Trematoda of the order Digenea and has a wide worldwide distribution [21]. From the Cestoda class, belonging to the Anoplocephalidae family, the species *Anoplocephala magna*, *Anoplocephala perfoliata* and *Paranoplocephala mamillana* are known to be relevant to horses [22].

The severity and clinical signs caused by helminth parasite infections will depend on the degree of parasite infection and the immunity of the affected animal [20]. These include anemia, fever, loss of appetite, weight loss, apathy, colic, dull hair, growth retardation, diarrhea and the possibility of death [23,24].

Climatic conditions have a significant impact on the helminth burden in horses, directly influencing the development of larvae in pastures. During spring and summer, high temperatures and rainfall favor greater proliferation of larvae, resulting in greater contamination of the environment and, consequently, in horses. In the dry season, the opposite happens. The water period is characterized by an increase in helminth infection rates, which makes strategic helminth control even more necessary [25,26].

### 3.2. Anthelmintic Resistance

The antiparasitic drugs used to control helminths in horses are predominantly grouped into four chemical classes: imidazothiazoles, benzimidazoles, pyrimidines and macrocyclic lactones. Rotation between these classes is widely adopted by breeders to minimize the impact of helminths on animal health and mitigate the risk of parasite resistance [27]. Among these, macrocyclic lactones are recognized as the most widely used class of anthelmintics [28].

The increasing indiscriminate use of these anthelmintics has contributed to the emergence of anthelmintic-resistant nematode populations, especially those belonging to the Cyathostominae subfamily, seriously threatening equine health, welfare and production in various locations around the world [29,30,31,32,33].

The current global situation is one of resistance to most of the classes of commercial anti-parasitic drugs available on the market. The worldwide occurrence of anthelmintic resistance to cyathostomes and *Parascaris equorum* is of great concern, as resistance has been reported to all currently available drug classes. Resistance in cyathostomes to benzimidazoles has been reported in 14 countries. Although less common, resistance to pyrantel in cyathostomes has been described in 12 countries [34].

The administration of anthelmintics, often carried out without the application of adequate technical criteria, occurs in an empirical and indiscriminate manner, which can compromise the effectiveness of these products, causing anthelmintic resistance to emerge and spread [35,36]. Drug resistance is hereditary, and repeated dosing will therefore select an increasing proportion of resistant parasites. The mechanisms involve differences in the metabolism of the anthelmintic within the parasite and/or mutations in the binding site of the drug molecule [37].

It is therefore extremely important to develop alternative strategies of parasite control, promoting a reduction in the number of infective larvae in pastures. This will reduce the reinfection of animals and, consequently, decrease the use of anthelmintics [2,38].

### 3.3. Helminthophagous Fungi

#### 3.3.1. Virulence Mechanisms

More than 150 species of helminthophagous fungi have been catalogued. These fungi are also known as helminth destroyers [39]. Research using fungi to control nematodes has been gradually increasing [38,40,41,42,43]. The fungus *Duddingtonia flagrans*, presented in the Bioverm^®^ product, has demonstrated efficacy in the biological control of gastrointestinal nematodes in horses kept on pasture over a period of six months [42]. According to [43], after oral administration of Bioverm, which contains the fungi *Duddingtonia flagrans* and *Pochonia chlamydosporia*, a significant reduction was observed in the number of infective larvae in the pasture and in the parasite load of treated horses, compared to untreated animals.

From an ecological point of view, the interaction between these fungi and nematodes can contribute to the maintenance and stability of the infective forms of helminths present in pastures [44,45].

According to [46], helminthophagous fungi can be divided into five groups: predators, opportunists or ovicides, endoparasites, toxin producers and producers of special attack devices. The fungi in the first group produce modified hyphae called traps, with which they bind and digest nematode larvae by a mechanical/enzymatic process. Fungi belonging to the ovicidal group produce hyphae, appressoria and enzymes that digest helminth eggs. Thus, they are the two groups that work best in preying on helminth parasites of animals [2,47]. On the other hand, the enzymatic activity of these organisms has sparked great interest in studies across different countries. For example, in China, a recombinant protein from *Arthrobotrys oligospora*, one of the widely studied nematode-capturing fungi, has been shown to have high chitinase activity, and it can degrade the eggshell of the following nematode species: *Strongylus equinus*, *Caenorhabditis elegans* and *Haemonchus contortus*, as well as the eggshell of the trematodes *F. hepatica* and *Dicrocoelium chinensis* [48].

Helminthophagic fungi act exogenously [49]. In other words, the presence of pre-parasitic stages (eggs and/or larvae) of helminths induces morphogenesis and the expression of virulence genes in these fungi, signaling the transition from the saprophytic stage to the phagocytic stage [2,8].

Predatory fungi develop specialized structures along their hyphae. The structures, with the function of capturing the nematodes, can be in the form of adhesive nets, adhesive buttons, constrictor and non-constrictor rings [50]. The formation of these traps occurs in response to the presence of the nematode or its excreta, biological compounds, or it can be induced by physiological stress conditions [47,51,52]. In addition to forming these specialized structures, predatory fungi produce chlamydospores interspersed with the hyphae [53]. Chlamydospores are resistant structures that guarantee the fungus’ great robustness [54] by stimulating its survival until the environmental factors are ideal for its development. It is the most widely researched group of fungi in the biological control of nematodes and has the greatest potential for industrialization [55].

The appearance of predatory structures occurs 30 min to 4 h after the fungus–nematode interaction. In the case of infective nematode larvae, the external cuticle can delay the infection process, but it does not provide protection against the predatory action of the fungi [56].

Opportunistic fungi are able to colonize nematode eggs and females through their appendages, entering the infective stage of the nematode [57]. The enzymes chitinases and proteases play an important role during the penetration of the eggshell, causing the destruction of its layers, and are therefore referred to as ovicides [55]. Among the most studied genera of this group of fungi are *Purpureocillium*, *Mucor circinelloides* and *Pochonia chlamydosporia* [8].

When present in the environment, they produce an extensive system of hyphae, thus forming traps that capture nematodes mechanically or by adhesion. They behave as natural antagonists, promoting seizure and the destruction of the nematode. According to their mechanism of action, they are classified as endoparasites, predators and opportunists, and the predator and opportunist groups have been studied for the biological control of helminthiases, with promising results [2,8,10,49,58].

The level of oxygen in the medium, as well as the substrate that makes up this culture medium, which influences a climatic environment that is favorable to the development of different stages of helminths, also makes it possible for fungi to survive, allowing them to prey on parasitic agents [39,55,59,60]. On the other hand, in the environment, these organisms can survive as saprophytic agents, feeding on environmental organic matter, or as parasites, feeding on a wide variety of helminths at all life stages, until they reach their infective stage [61,62].

As a characteristic shared by all types of helminthophagous fungi, recognition of the host and adherence to the cuticle of the infective larvae and/or eggs of the target organisms are the initial steps in infection [8].

#### 3.3.2. Survival Mechanisms

The germination rate, sporulation and resistance of *Duddingtonia flagrans* chlamydospores are influenced by factors such as temperature, pH, ultraviolet radiation, osmotic pressure and the availability of carbon, nitrogen and oxygen in the soil. At temperatures around 25 °C and pH 7–9, the germination rate of chlamydospores reaches its maximum threshold, which favors mycelial production [63].

Another essential requirement for a fungal isolation to be possibly exploited in the biological control of parasites is to resist passage through the gastrointestinal system tract of equines after oral administration. In other words, it must tolerate stressful conditions and, once present in the feces, it must be able to germinate, colonize the fecal bolus and capture the larval stages hatched from the eggs before they migrate out of the fecal bolus [64]. In addition, it must have specificity of action, high reproductive capacity and withstand the environmental and climatic conditions of the geographical region where the control will be carried out [2].

Ref. [65] observed that *Duddingtonia flagrans*, *Arthrobotrys cladodes* and *Pochonia chlamydosporia* were not limited by temperature variations between 15 and 35 °C for nematode control under in vitro conditions, although they showed greater growth, chlamydospore production and nematicidal activity at intermediate temperatures (20, 25 and 30 °C).

#### 3.3.3. Overview of Helminthophagous Fungi Species

*Duddingtonia flagrans* is a predator of L3 larvae of gastrointestinal parasitic nematodes and stands out as one of the most studied agents for use in biological control [2], acting by means of three-dimensional adhesive networks formed by its hyphae to capture the infective nematode larvae present in the environment, especially in animal feces. After capturing the larvae, the fungus penetrates it and uses enzymes to digest its internal contents, obtaining essential nutrients for its growth and reproduction. The formation of chlamydospores, resistant structures that protect the fungus in adverse conditions, allows it to survive as it passes through the gastrointestinal tract of horses. Once excreted in the feces, the chlamydospores germinate, forming capture nets that significantly reduce the number of infective larvae in the environment, reducing contamination and preventing reinfections [7,55].

It has significant potential for controlling Cyathostominae in laboratory conditions, and it is a promising biological agent for managing cyathostomin infections in horses [2,42,66,67]. Studies have shown that the fungus *Duddingtonia flagrans*, present in Bioverm^®^, contributed to the biological control of gastrointestinal nematodes in horses when tested under field conditions for a period of six months [42]. According to [43], after oral administration of Bioverm, which contains the fungi *Duddingtonia flagrans* and *Pochonia chlamydosporia*, reductions were observed in the number of infective larvae in the pasture and in the parasite load of horses compared to untreated animals.

This species has stood out due to its diversity of mechanisms of action and large production of chlamydospores [7]. However, *Duddingtonia flagrans* has shown other interesting aspects in its mechanism of action, since in addition to the traps, it has good enzyme production and nanoparticles, making it a species with a broad range of action, to be better explored for biological control [68,69].

The development of appressoria formed from undifferentiated hyphae, which colonize and penetrate the eggshell causing its destruction, is a characteristic of the *Pochonia chlamydosporia* species [55,58,69,70]. This fungus can produce enzymes with possible biotechnological applications [71]. The enzymes proteases, chitinases and lipases are necessary during the infection process, since the protective barriers of the nematode, cuticle and eggshell, are rich in macromolecules that are the substrates for these enzymes: proteins, chitin and lipids [71,72,73]. It was shown that *Pochonia chlamydosporia* maintained its viability and ability to attack *Oxyuris equi* eggs passing through the gastrointestinal tract of horses, demonstrating the viability of its application in biotechnology for parasite management [64].

The hyphae-forming structures of the fungi *Pochonia chlamydosporia* and *Purpureocil-lium* lilacinum embed themselves in the shells of trematode, ascarid and trichurid eggs, penetrating and destroying the embryo inside them [74,75].

The species *Mucor circinelloides* is also capable of adhering to the surface of helminth eggs, penetrating and feeding on their contents [76]. It has saprophytic capacity, acting against trematode eggs (*Fasciola hepatica, Calicophoron daubneyi*) [77], ascarids (*Toxocara canis*, *Toxascaris leonina*, *Ascaris suum, Bayliscascaris procyonis*) and trichurids (*Trichuris* spp.) [67,78]. Studies report that *Mucor circinelloides* can be associated with *Duddingtonia flagrans*, with a very practical ovicidal and larvicidal activity for the control of helminths, whose infective stages are eggs or larvae that develop in the environment [79].

#### 3.3.4. Methods of Administering Helminthophagous Fungi

**Figure 1:**
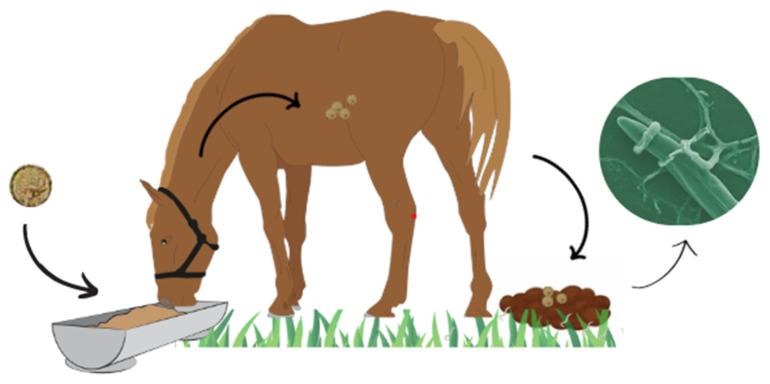
Dynamics of helminth control in horses using helminthophagous fungi.

The most widely used form of helminthophagous fungi in field conditions is through feed supplementation [42,43] (Figure 1).

It is through the oral administration of these fungal isolates in animal feed that the so-called ‘classical biological control’ takes place, a term that can refer to the flooding of these biological agents into the soil [55]. In the fecal pat, the development of these fungi is stimulated by contact with an increasing number of free-living larvae. With colonization, the chlamydospores establish themselves in the pasture, followed by subsequent ingestion of the pasture by the animals [79]. The parasite load present in the pasture, i.e., when the helminths are not in their parasitic stage, represents around 95% of the total parasite population. As such, almost all of this load is found in the environment, and the fungi therefore act on the site of greatest relevance for control, reducing the number of third-stage larvae in the pastures and, consequently, reducing the infection rate [80,81].

#### 3.3.5. Commercial Products—Biotechnological Innovation

**Figure 2:**
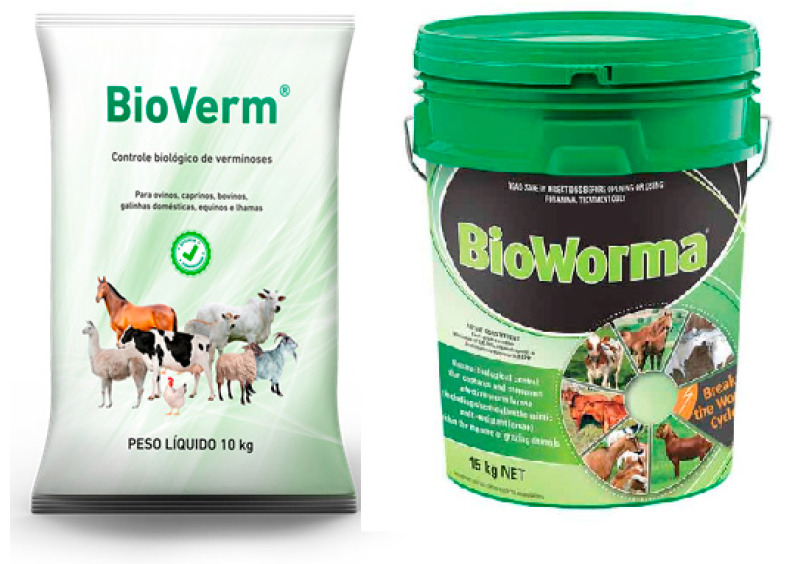
Commercial formulations containing *Duddingtonia flagrans*: BioVerm^®^ (Brazil) and BioWorma^®^ (Australia). Source: Ghenvet and IAHP.

Recently, commercial products with formulations containing *Duddingtonia flagrans* have started to become available. In Brazil (Bioverm-AC001, GhenVet Saúde Animal, Paulínia, Brazil), in Australia and in New Zealand (BioWorma-NCIMB 30336, BioWorma, Sydney, Australia), these products are already in use, with administration in animal feed [41,42,43,82,83,84] (Figure 2).

Bioproducts formulated from helminthophagous fungi are found in different forms of administration. Some examples are found in the form of sodium alginate pellets, controlled administration cakes and homogenized cereal grains. The species that have already been successfully incorporated are *Monacrosporium thaumasium*, *Arthrobotrys robusta* and *Duddingtonia flagrans*. It is important to note that these formulations are preconditioned to withstand passing through the animals’ gastrointestinal tract without losing the viability of the fungi [7].

The administration interval of the formulations, as well as the dose to be given to the animals, seems to be crucial to guarantee the effectiveness of any biological control method [81]. After the administration of *Duddingtonia flagrans* is stopped, its effect on the population of nematode larvae decreases rapidly. Therefore, to achieve a significant and sustained reduction in infective larvae in pastures, it is essential to provide daily and continuous doses of the fungus for prolonged periods. This continuous strategy maintains the effectiveness of biological control, reducing the environmental parasite load and, consequently, the reinfection of animals [40].

Refs. [85,86] evaluated the ability of chlamydospores from the ovicidal fungus *Mucor circinelloides* and the larvicidal *Duddingtonia flagrans* to withstand the industrial pelleting process. The fungal spores were added in the process of mixing the feed components, which were then subjected to a temperature of 72 °C. The chlamydospores of both fungi were able to withstand the thermal challenge of the pelleting process, maintaining their ability to infect and destroy nematode eggs. When nutritional pellets containing chlamydospores of the fungi *Mucor circinelloides* and *Duddingtonia flagrans* (both incorporated into the same pellet) were fed to horses, a significant reduction in cyathostome egg counts was recorded, and no adverse effects were observed after administration, nor were any changes recorded in the values of cellular blood parameters [85,87].

The oral administration of grains containing nematophagous fungi previously cultivated and provided to animals to evaluate the reduction in infective forms of parasites in feces is also efficient [80,83], and it has been shown to be a commercially practical way [41].

Recently, a new possibility of using these fungi in nematode control was launched, by means of nanoparticles (NPs) biosynthesized from fungal filtrates [88]. The nanoparticles biosynthesized by *D. flagrans* were shown to be capable of destroying infective larvae of *Ancyostoma caninum* [89,90]. In the study by [91,92], it was observed that the extracellular enzymes produced by *Duddingtonia flagrans* show nematocidal activity against the infective larvae of cyathostomins. This indicates that enzymes such as chitinase play a fundamental role in the biological control of nematodes, acting directly on the third-stage larvae (L3), which is the infective form. Reference [69] investigated the effect of silver nanoparticles (AgNPs) from *Duddingtonia flagrans* on cyathostomin larvae. The study showed that AgNPs have nematicidal activity, specifically on infective Cyathostominae larvae. This discovery has opened up new ways for the use of this fungus and, therefore, new products, such as bio-antihelminthics may, in the future, serve as an alternative for more successful parasite control against the already established problem of parasite resistance [7].

Finally, an interesting way of administration involved the preparation of edible gelatins containing a mixture of chlamydospores of *Mucor circinelloides* and *Duddingtonia flagrans*. This formulation provided excellent results in reducing the risk of infection by trichostrongylids, roundworms, whipworms and hookworms affecting dogs and wild captive herbivores [9,93].

### 3.4. Biological and Sustainable Solution

Gastrointestinal nematode anthelmintic resistance has become a growing problem worldwide, especially due to the indiscriminate and empirical use of anthelmintics, without adequate technical criteria, exacerbating the resistance problem globally [32,33,94]. In addition, anthelmintics such as macrocyclic lactones (e.g., ivermectin and doramectin) are excreted in animal feces in large quantities without undergoing significant modifications, with more than 90% of the molecules being eliminated as the parent drug. This can negatively affect the community of invertebrates and microorganisms that depend on feces for their development and food, impacting the decomposition of feces and soil quality. The environmental effect of these residues is well documented in cattle [95]. In addition, the use of these drugs can leave residues in animal products such as meat, milk and eggs, which must be below the limits established to guarantee food safety, known as “maximum residue limits” [96].

The lack of adequate treatment of helminthoses in horses can result in serious gastrointestinal disorders, compromising the health and development of the animals, which can lead to significant losses, including death [97,98]. In this context, the use of nematophagous fungi, such as *Duddingtonia flagrans* and *Pochonia chlamydosporia*, has proven to be a viable and effective alternative. Unlike anthelmintics, these fungi do not negatively affect the microbial population of feces, which hinder the development of beneficial microorganisms [43]. Studies have shown that the oral administration of *Duddingtonia flagrans* does not cause any adverse effects on the health of horses, as there is no action on the animal during the passage of these fungi through the animal’s gastrointestinal tract, making nematophagous fungi a safe and sustainable option for nematode control compared to traditional chemical treatments [87,99]. In another study, ref. [100] showed that *Duddingtonia flagrans* did not cause damage or alterations to intestinal microvilli in Swiss mice infected with *Strongyloides venezuelensis* after oral ingestion of chlamydospores/conidia. In addition, it did not interfere with the levels of eosinophil peroxidase and myeloperoxidase, providing promising information for future research focused on the in vivo use of these enzymes. Thus, the use of nematophagous fungi represents an effective approach that is less harmful to the environment and safe for the health of animals and consumers.

## 4. Conclusions

The application of helminthophagous fungi in the biological control of gastrointestinal nematodes in horses is a promising and sustainable alternative to the challenges faced by equine farming, especially in relation to the presence of anthelmintic resistance caused by the indiscriminate use of chemical anthelmintics. Species such as *Duddingtonia flagrans*, *Pochonia chlamydosporia*, *Arthrobotrys oligospora*, *Monacrosporium thaumasium*, *Mucor circinelloides* and *Purpureocillum liacinum* have shown efficacy in reducing the parasite burden by acting on different stages of the nematode life cycle, from the larval stage to the egg stage.

The administration of these fungi is practical and can be carried out by means of feed supplementation or commercial formulations that guarantee the survival of the chlamydospores during their passage through the animals’ digestive tract. By being excreted in the feces, these fungi act directly on the environment, reducing the amount of infective larvae in pastures and reducing the reinfection of horses.

In view of this, the use of helminthophagous fungi can be considered a viable, efficient and ecologically correct strategy for the control of helminthiases in horses, offering a practical and long-lasting solution for anti-parasitic management.

## Data Availability

Data sharing not applicable to this article as no datasets were generated or analyzed during the current study.
